# Effect of colony morphology variation of *Burkholderia pseudomallei *on intracellular survival and resistance to antimicrobial environments in human macrophages in vitro

**DOI:** 10.1186/1471-2180-10-303

**Published:** 2010-11-30

**Authors:** Sarunporn Tandhavanant, Aunchalee Thanwisai, Direk Limmathurotsakul, Sunee Korbsrisate, Nicholas PJ Day, Sharon J Peacock, Narisara Chantratita

**Affiliations:** 1 Department of Microbiology and Immunology, Faculty of Tropical Medicine, Mahidol University, Bangkok, Thailand; 2 Mahidol-Oxford Tropical Medicine Research Unit, Faculty of Tropical Medicine, Mahidol University, Bangkok, Thailand; 3 Department of Immunology, Faculty of Medicine Siriraj Hospital, Mahidol University, Bangkok, Thailand; 4 Department of Tropical Hygiene, Faculty of Tropical Medicine, Mahidol University, Bangkok, Thailand; 5 Center for Clinical Vaccinology and Tropical Medicine, Nuffield Department of Clinical Medicine, University of Oxford, Churchill Hospital, Oxford, UK; 6 Department of Medicine, University of Cambridge, Addenbrooke's Hospital, Cambridge, UK

## Abstract

**Background:**

Primary diagnostic cultures from patients with melioidosis demonstrate variation in colony morphology of the causative organism, *Burkholderia pseudomallei*. Variable morphology is associated with changes in the expression of a range of putative virulence factors. This study investigated the effect of *B. pseudomallei *colony variation on survival in the human macrophage cell line U937 and under laboratory conditions simulating conditions within the macrophage milieu. Isogenic colony morphology types II and III were generated from 5 parental type I *B. pseudomallei *isolates using nutritional limitation. Survival of types II and III were compared with type I for all assays.

**Results:**

Morphotype was associated with survival in the presence of H_2_O_2 _and antimicrobial peptide LL-37, but not with susceptibility to acid, acidified sodium nitrite, or resistance to lysozyme, lactoferrin, human neutrophil peptide-1 or human beta defensin-2. Incubation under anaerobic conditions was a strong driver for switching of type III to an alternative morphotype. Differences were noted in the survival and replication of the three types following uptake by human macrophages, but marked strain-to strain-variability was observed. Uptake of type III alone was associated with colony morphology switching.

**Conclusions:**

Morphotype is associated with phenotypes that alter the ability of *B. pseudomallei *to survive in adverse environmental conditions.

## Background

*Burkholderia pseudomallei *is an environmental Gram-negative bacterium that causes a severe and often fatal disease called melioidosis. This is an important cause of sepsis in south-east Asia and northern Australia, a geographic distribution that mirrors the presence of *B. pseudomallei *in the environment [1]. Melioidosis may develop following bacterial inoculation or inhalation and occurs most often in people with regular contact with contaminated soil and water [1]. Clinical manifestations of melioidosis are highly variable and range from fulminant septicemia to mild localized infection. The overall mortality rate is 40% in northeast Thailand (rising to 90% in patients with severe sepsis) and 20% in northern Australia [1,2].

A major feature of melioidosis is that bacterial eradication is difficult to achieve. Fever clearance time is often prolonged (median 8 days), antimicrobial therapy is required for 12-20 weeks, and relapse occurs in around 10% of patients despite an appropriate course of antimicrobial therapy [3,4]. The basis for persistence in the infected human host is unknown, although several observations made to date may be relevant to the clinical behaviour of this organism [2,5]. *B. pseudomallei *can resist the action of bactericidal substances including complement and antimicrobial peptides in human serum [6-8]. *B. pseudomallei *can also survive after uptake by a range of phagocytic and non-phagocytic cells. Macrophages have several strategies to control bacterial infection, including bacterial killing following uptake through the action of reactive oxygen and reactive nitrogen compounds, antimicrobial peptides and lysozomal enzymes. Despite this, *B. pseudomallei *can invade and replicate in primary human macrophages [8-10].

Bacterial survival under adverse and rapidly changing environmental conditions is likely to be facilitated by phenotypic adaptability and plasticity. A previous study conducted by us found that 8% of primary cultures of clinical samples taken from patients with melioidosis contained more than one colony morphotype on Ashdown agar. Morphotypes could switch reversibly from one to another under specific conditions, and were associated with variable expression of putative virulence determinants including biofilm and flagella [11]. Compared with parental type I (the common 'cornflower head' morphology), isogenic type II (a small, rough colony) had increased biofilm and protease production, while isogenic type III (a large, smooth colony) was associated with increased flagella expression [11]. *In vitro *models suggested that switching of morphotype impacted on intracellular replication fitness after uptake by human epithelial cell line A549 and mouse macrophage cell line J774A.1. We postulated that colony morphology switching might represent a mechanism by which *B. pseudomallei *can adapt within the macrophage and persist *in vivo*.

In this study, we investigated whether the variable phenotype associated with different morphotypes resulted in altered fitness during interactions with the human macrophage cell line U937 and after exposure to a range of laboratory conditions that simulate one or more conditions within the macrophage milieu. Isogenic morphotypes II and III generated from each parental type I of 5 *B. pseudomallei *strains isolated from patients or soil were used in all experiments.

## Results

### Growth curve analysis of isogenic morphotypes

Different growth rates may affect the number of intracellular bacteria following uptake by host cells. Thus, prior to observation of intracellular replication in macrophages, extracellular growth of *B. pseudomallei *was compared between 3 isogenic morphotypes cultured in trypticase soy broth (TSB). Using a starting inoculum of 1 × 10^4 ^CFU/ml, log and stationary phase occurred at 2 h and 12 h, respectively, for all 3 morphotypes. There was no difference in doubling time between 3 isogenic morphotypes (*P *= 0.14) with an average doubling time of 40.2, 39.2 and 38.3 minutes for types I, II and III, respectively.

### Replication of isogenic *B. pseudomallei *morphotypes in macrophages

Evaluation of the initial *B. pseudomallei*-macrophage cell interaction using a multiplicity of infection (MOI) of 25:1 demonstrated that 3.0% of the bacterial inoculum (range 1.2-8.0% for different isolates) was associated with macrophages at 2 h. There was no significant difference in this value between 3 isogenic morphotypes for all 5 isolates.

Following removal of extracellular bacteria and incubation for a further 2 h, 1.5% of the bacterial inoculum (range 0.4-3.4% for different isolates) was recovered. There was no significant difference in this value between 3 isogenic morphotypes for all 5 isolates.

**Figure 1:**
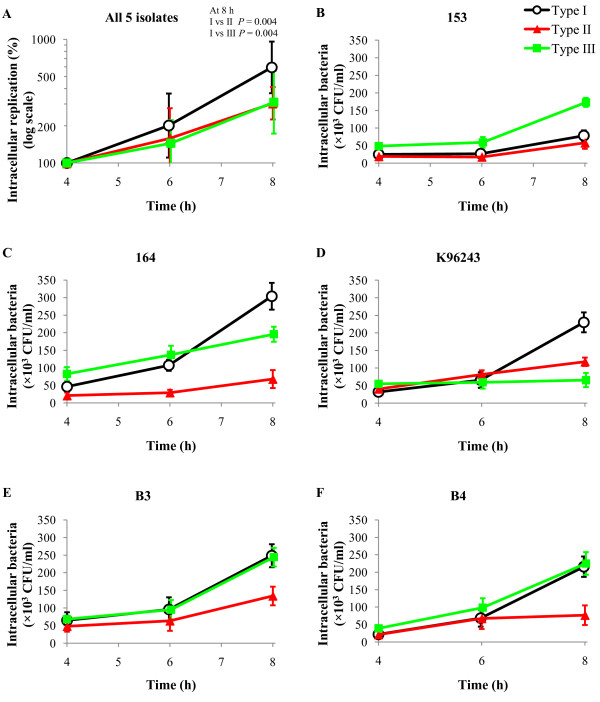
**Intracellular replication of 3 isogenic morphotypes of *B. pseudomallei *in human macrophages**. Differentiated U937 cells were incubated for 2 h with *B. pseudomallei *at a MOI of 25:1, after which non-adherent bacteria were removed by washing and incubation for a further 2 h with kanamycin. At this 4 h time point, fresh medium containing kanamycin was added and incubation continued for a further 4 h. The bacterial count and colony morphology were enumerated at 4, 6 and 8 h by cell lysis and plating onto Ashdown agar. The data shown in Figure 1A represent mean values for each isogenic morphotype derived from 5 *B. pseudomallei *isolates and is expressed as the bacterial proportion at 6 and 8 h compared with the number at 4 h (which was defined as 100%). Figure 1B-1F shows the number of intracellular bacteria in CFU/ml for individual isolates. Data plots are means ± standard deviations.

The intracellular replication of *B. pseudomallei *between 4 to 8 h within macrophages is summarized in Figure 1. The replication rates for the 3 isogenic morphotypes of each strain obtained from two independent experiments were comparable (data not shown). Percent replication at 8 h was defined in relation to the 4 h time point, which was used as the reference count. Analysis of pooled data for 5 isolates demonstrated that type I had a significantly higher rate of intracellular replication than either type II or III. The mean intracellular replication of type I at 8 h was 2.0 (95%CI 1.5-2.6, *P *= 0.004) times higher than that of type II, and 1.9 (95%CI 1.4-2.5, *P *= 0.004) times higher than that of type III (Figure 1A). However, this pattern was not uniformly observed for each of the 5 isolates, as shown in Figure 1B-F. The higher replication fitness for type I based on the summary data was largely accounted for strains 164 and K96243. Other strains demonstrated a different pattern. For example, strain 153 type III had a higher intracellular replication than type I, a finding that replicates those of a previous study [11]. The mean intracellular bacterial count also varied between individual isolates. These differences were not due to the relative sensitivities of 3 isogenic morphotypes to 250 μg/ml kanamycin, as this experimental condition removed 99.9% of extracellular bacteria independent of type for all isolates (data not shown).

### Susceptibility of isogenic morphotypes to acid

To examine the effect of acid, growth of 3 isogenic morphotypes in LB at pH 4.0, 4.5, 5.0 and 7.0 was compared at each of 5 time points over 24 h of incubation. No growth difference was observed between morphotypes at any time point for pH 4.5, 5.0 or 7.0 (*P *> 0.10 for all time points). When cultured in LB broth at pH 4.0, all bacteria died within 12 h incubation.

### Susceptibility of isogenic morphotypes to reactive oxygen intermediates (ROI)

**Figure 2:**
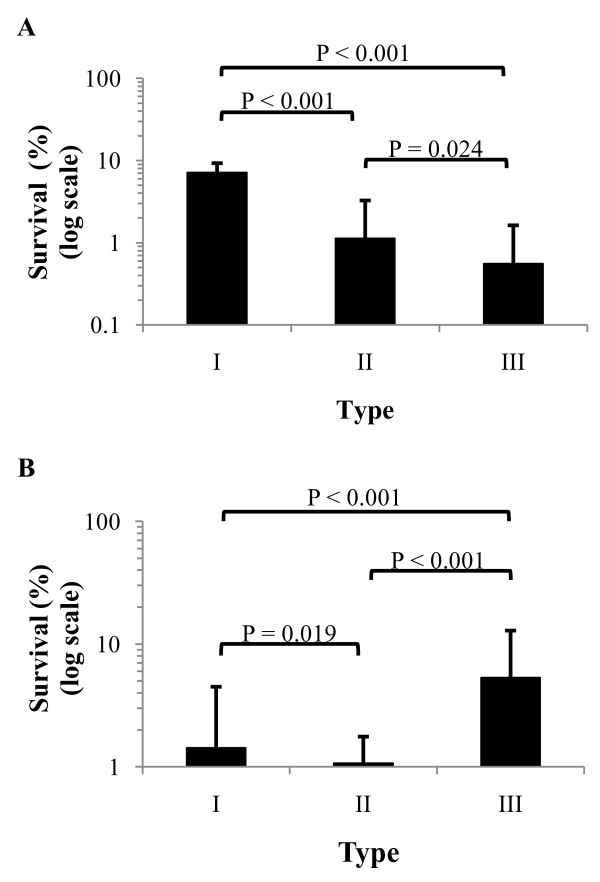
**Susceptibility of 3 isogenic morphotypes of *B. pseudomallei *to ROI and antimicrobial peptide LL-37**. Survival was examined for 5 different *B. pseudomallei *isolates. (A) Percent survival in ROI was determined on LB agar plates containing 625 μM H_2_O_2 _compared to the number of bacteria on plates without H_2_O_2_. The results were obtained from 4 separate experiments. (B) Percent survival in LL-37 was determined at 6.25 μM LL-37 in 1 mM potassium phosphate buffer (PPB) pH 7.4 for 6 h. The results were obtained from 2 separated experiments. Data plots are means ± standard deviations.

The susceptibility of 3 morphotypes to ROI was initially examined on LB agar plates containing a range of H_2_O_2 _concentrations (0, 170, 310, 625, 1,250 and 2,500 μM) (data not shown). *B. pseudomallei *failed to grow on plates with H_2_O_2 _at a concentration higher than 625 μM, and so the percentage of viable bacteria were enumerated using agar plates with 625 μM H_2_O_2 _compared to those on plates without H_2_O_2_. This demonstrated a difference in bacterial survival between the three isogenic morphotypes (*P *< 0.001). Percentage survival of type I was 3.8 (95%CI 2.9-5.0, *P *< 0.001) times higher than that for type II, and was 5.2 (95%CI 4.0-6.8, *P *< 0.001) times higher than that for type III (Figure 2A).

Further examination was undertaken of the susceptibility of the 3 morphotypes with a range of concentrations of H_2_O_2 _in LB broth. No bacteria survived in 500 μM and 250 μM H_2_O_2_. In 125 μM H_2_O_2_, type I of all 5 isolates multiplied from 1 × 10^6 ^CFU/ml (the starting inoculum) to between 5 × 10^7 ^and 2.1 × 10^8 ^CFU/ml. By contrast, all 5 type III and 4 type II isolates (the exception being type II derived from isolate 164) obtained from the same experiment demonstrated no growth on the plates. This confirmed a higher resistance to H_2_O_2 _of parental type I compared to types II and III. A difference was also observed between three isogenic morphotypes in 62.5 μM H_2_O_2 _(*P *< 0.001). Bacterial growth of type I was 1.5 (95%CI 1.1-2.0, *P *= 0.02) times higher than that for type II, and was 2.7 (95%CI 2.0-3.7, *P *< 0.001) times higher than that for type III.

### Susceptibility of isogenic morphotypes to reactive nitrogen intermediates (RNI)

Susceptibility of *B. pseudomallei *to RNI was observed following 6 h exposure to various concentrations of NaNO_2 _ranging between 0.1 to 10 mM in acidified pH 5.0 in LB broth. Using a concentration of 2 mM NaNO_2_, the percent survival of types I, II and III were 43.8%, 43.7% and 40.1%, respectively, with no difference observed between the three morphotypes (*P *> 0.10).

### Susceptibility of isogenic morphotypes to lysozyme and lactoferrin

Compared with initial inocula and untreated controls, treatment with 200 μg/ml lysozyme at pH 5.0 did not decrease the bacterial count for the 3 isogenic morphotypes of *B. pseudomallei*, while this concentration could reduce the number of *E. coli *from 4.9 × 10^6 ^CFU/ml (the starting inoculum) to 425 CFU/ml. Susceptibility was examined further in the presence of 3 mg/ml lactoferrin. A kinetic study over time demonstrated that lactoferrin alone could kill an entire *E. coli *inoculum of 1 × 10^6 ^CFU/ml within 3 h at pH 5.0. The same treatment did not affect the number of viable *B. pseudomallei *which was comparable to the inoculum and untreated control. Adding 200 μg/ml lysozyme with lactoferrin did not enhance the killing efficacy of *E. coli *and had no effect on *B. pseudomallei*.

### Susceptibility of isogenic morphotypes to antimicrobial peptides

Macrophages produce several antimicrobial peptides [12,13]. We examined the susceptibility of isogenic morphotypes to HNP-1, HBD-2 and cathelicidin LL-37, three of the main human antimicrobial peptides. The results demonstrated that 100 μg/ml HNP-1 and 100 μg/ml HBD-2 did not reduce the bacterial count for the 3 isogenic morphotypes of any of the *B. pseudomallei *isolates when compared with the initial inocula and untreated controls.

In a pilot experiment with a range of LL-37 concentrations and exposure times, we found that LL-37 reduced the *B. pseudomallei *count at a concentration of 6.25 μM at 6 h. This condition killed 100% of a starting inoculum of 4.6 × 10^6 ^CFU/ml *E. coli *control and caused a 75.7 to 99.8% reduction of *B. pseudomallei *for different isolates. A difference in bacterial survival was observed between the three isogenic morphotypes (*P *< 0.001). Survival of type I was 1.5 (95%CI 1.1-2.2, *P *= 0.02) times higher than that for type II, but was 3.7 (95%CI 2.6-5.3, *P *< 0.001) times lower than that for type III (Figure 2B).

### Growth in low oxygen concentrations

Low oxygen concentration may limit the intracellular growth of aerobic bacteria within the host [14]. We examined the survival of 3 isogenic morphotypes and determined whether morphotype switching occurred in response to different oxygen concentrations during incubation on Ashdown agar at 37°C. *B. pseudomallei *survived in 5-15% oxygen concentration for 14 days, with an average colony count of 95% (range 72-109% for different isolates and morphotypes) compared to control plates incubated in air for 4 days (Table 1). There was no difference in the survival pattern between 3 isogenic morphotypes (*P *> 0.10). *B. pseudomallei *colonies were not visible on Ashdown agar after incubation in an anaerobic chamber for 2 weeks. The capability to recover from anaerobic conditions was observed as colonies were visible at 48 h after reincubation at 37°C in air, and colony counts were performed after incubation for 4 days. The percentage of bacteria recovered was not different between three morphotypes (*P *> 0.10).

**Table 1:** Growth and morphotype switching of 3 isogenic morphotypes derived from 5 *B. pseudomallei* isolates following incubation in low oxygen and anaerobic conditions

	Atmospheric conditions during incubation at 37°C								
	
Starting type	Air for 4 days (control)			5-15% oxygen for 14 days			Reincubated in air for 4 days following anaerobic conditions for 14 days		
	
	Mean colony count, (range)	*Morphotype, % (range)		Mean % colony count compared with control in air (range)	*Morphotype, % (range)		Mean % colony count compared to control in air (range)	*Morphotype, % (range)	
I (parental)	101 (93-106)	I	100%	92% (78-108%)	I	100%	86% (57-138%)	I	100%

II	90 (62-150)	II	100%	91% (72-109%)	II	100%	95% (66-127%)	II	100%

III	123 (110-141)	III	89% (81-98%)	98% (78-107%)	III	89% (81-99%)	80% (48-94%)	III	17% (0-85%)
		I or II	11% (2-19%)		I or II	11% (1-19%)		I or II	83% (15-100%)

The data represents the mean of 5 *B. pseudomallei *isolates for each morphotype. The range reflected variation of % colony count between isolates. *% Morphotype was the proportion of each morphotype on the plate. Morphotype switching was observed for type III (starting type) to either type I (isolates K96243, 164, B3 and B4) or to type II (isolate 153).

### Effect of laboratory conditions on morphotype switching

**Figure 3:**
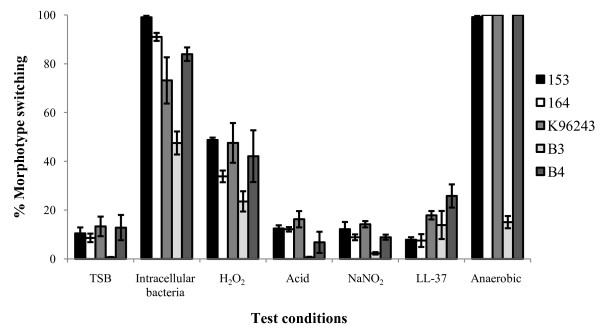
**Effect of seven conditions on morphotype switching of type III of 5 *B. pseudomallei *isolates**. (i) TSB culture in air with shaking for 28 h; (ii) intracellular replication in macrophages for 8 h; (iii) exposure to 62.5 μM H_2_O_2 _in LB broth for 24 h; (iv) growth in LB broth pH 4.5 for 24 h; (v) exposure to 2 mM NaNO_2 _in LB broth for 6 h; (vi) exposure to 6.25 μM LL-37 in 1 mM potassium phosphate buffer (PPB) pH 7.4 for 6 h; and (vii) re-exposure to air after incubation in anaerobic chamber for 2 weeks. All experiments were performed using the experimental details described above. *B. pseudomallei *morphotype on Ashdown agar following incubation in air at 37°C for 4 days was defined and compared with the starting morphotype. Morphotype switching was presented as the proportion (%) of alternative types in relation to the total colonies present.

Types I and II did not demonstrate colony morphology variation over time in any of the conditions tested. Figure 3 shows the effect of various testing conditions of type III for all 5 isolates. Between 1% and 13% of colonies subcultured from 28 h TSB culture onto Ashdown agar switched to alternative types. The switching of type III appeared to be important for replication in macrophages. Following uptake, switching of type III increased over time such that by the 8 h time point, between 48-99% of the agar plate colonies (the range representing differences between isolates) had switched to type I (isolates K96243, 164, B3 and B4) or to type II (isolate 153). Morphotype switching did not increase in acid, acidified sodium nitrite, or LL-37. In contrast, morphotype switching from broth culture containing 62.5 μM H_2_O_2 _increased over time of incubation, ranging between 24-49% of the plate colonies for different isolates. Interestingly, between 15-100% of the total type III colony count switched to an alternative morphotype after recovery from anaerobic conditions. The pattern of morphotype switching in all conditions tested was specific to isolates, with four isolates switching from type III to type I (K96243, 164, B3 and B4), and one isolate switching to II (153).

## Discussion

Our previous paper reported a process of *B. pseudomallei *colony morphology switching that occurred during human melioidosis, and in an animal model, mouse macrophage cell line J774A.1, human lung epithelial cell line A549, and under starvation conditions *in vitro*. In this study, we investigated whether the variable phenotype associated with different morphotypes resulted in a survival fitness or disadvantage during interactions with a human macrophage cell line U937 and after exposure to factors that simulate the macrophage milieu. Although our previous report described 7 different morphotypes from clinical isolates, the five isolates used here from 3 different clinical and 2 environmental samples were only observed to switch under nutritional limitation from parental type I to types II and III, allowing comparison of 3 isogenic morphotypes with known variable phenotype.

The initial interaction between the human macrophage cell line U937 and 3 isogenic morphotypes of *B. pseudomallei *was not different between the three types. Despite a comparable rate of extracellular growth between isogenic morphotypes, heterogeneity in subsequent intracellular survival/growth after this time point was observed. Type III of each isolate was inconsistently capable of multiplication after uptake by human macrophages, and was associated with a change in morphotype. This suggests that type III has a fitness disadvantage under these circumstances. A possible explanation for this is that type III does not appear to produce biofilm [11]. A biofilm mutant demonstrated a mark reduction in intracellular survival in primary human macrophages than the wild type, suggesting that biofilm production is associated with the ability to survive in human macrophages [8].

Our previous study examined the survival and replication of *B. pseudomallei *strain 153 in the human respiratory epithelial cell line A549 and the mouse macrophage cell line J744A.1. Our finding here that type III of strain 153 had increased survival in the human macrophage cell line U937 is consistent with our previous findings for the mouse macrophage cell line J774A.1 infected with the same strain [11]. However, the use of a wider number of strains in this study demonstrated that there was a lack of reproducibility between strains. We suggest that this is likely relate to variability in genomic content between the strains tested. Future testing strategies require the evaluation of a large numbers of strains that have undergone whole genome sequencing to facilitate statistically robust comparisons between genomic variation and phenotypic behaviour.

Several components of the innate immune system are efficient in killing organisms within human macrophages [15]. The most important of these are the antimicrobial peptides and nitric oxide (NO), the superoxide anion (O_2_^-^), and hydrogen peroxide (H_2_O_2_), all of which are directly toxic to bacteria. Reactive oxygen species generated by the phagocyte NADPH oxidase have an essential role in the control of *B. pseudomallei *infection in C57BL/6 bone marrow derived macrophages [16]. Type I of all 5 *B. pseudomallei *isolates tested here had the greatest resistance to H_2_O_2_, followed by types II and III, respectively, suggesting that type I has the greatest potential to scavenge or degrade H_2_O_2 _molecules. This may explain the finding that type I had the highest replication after uptake by the macrophage cell line. Type III switched to type I or II during culture in medium containing H_2_O_2, _indicated that type III had a survival disadvantage under such conditions that required switching to a more H_2_O_2 _resistant type.

One of the mechanisms by which *B. pseudomallei *escapes macrophage killing is by repressing inducible nitric oxide synthase (iNOS) by activating the expression of two negative regulators, a suppressor of cytokine signaling 3(SOC3) and cytokione-inducible src homology2-containing protein (CIS) [17]. It is unknown whether there are variation between strains and isogenic morphotypes in the ability to interfere with iNOS induction. However, colony morphology differences did not influence resistance to RNI. *B. pseudomallei *is protected from RNI by the production of alkyl hydroperoxide reductase (AhpC) protein and depends on OxyR regulator and a compensatory *KatG *expression [18]. These mechanisms may not be associated with colony morphology variability.

*B. pseudomallei *survive in the phagolysosome [10] which are acidified environments containing lysozymes, proteins and antimicrobial peptides that destroy pathogen. There was no difference in growth for the 3 isogenic morphotypes of *B. pseudomallei *derived from all five isolates at all pH levels tested above 4.0, but a pH of 4.0 was universally bactericidal, suggesting that morphotype switching did not provide a survival advantage against acid conditions.

All morphotypes of *B. pseudomallei *were highly resistance to lysozyme and lactoferrin. Lysozyme functions to dissolve cell walls of bacteria. Lactoferrin is a competitor that works by binding iron and preventing uptake by the bacteria. Common structures for resistance to these factors such as capsule and LPS [8] were present in all isogenic morphotypes [11]. An alternative explanation is that *B. pseudomallei *may produce a morphotype-independent lysozyme inhibitor that counteracts the action of lysozyme and lactoferrin.

Antimicrobial peptides are efficient at killing a broad range of organisms. They are distributed in variety tissues, and in neutrophils and macrophages [12,13]. All 3 isogenic *B. pseudomallei *morphotypes were resistant to α-defensin HNP-1 and β-defensin HBD-2, but were susceptible to LL-37. In contrast to sensitivity to H_2_O_2_, type III was more resistant than type I or II to LL-37. This ability may allow type III to survive within host cells for a limited period before successfully switching to alternative phenotypes and may provide a fitness of advantage in macrophages.

Another feature of bacterial survival during the establishment of persistent infection in the host is adaptation to hypoxia in the host microenvironment [14]. This study demonstrated that all 3 isogenic morphotypes were able to tolerate a low oxygen concentration and anaerobic conditions for at least two weeks. Type III switching to either type I or II was observed during recovery from anaerobic incubation. The fact that types I and II were stable following anaerobic incubation suggests that they are tolerant of fluctuations in oxygen concentration.

Given the variation in the genome of different *B. pseudomallei*, it was not surprising to observe some variation in intracellular replication between isogenic morphotypes of different isolates. Only one strain switched from type III to II, while the other four isolates switched from type III to type I in all conditions in which a change in morphotype was observed. Analyses of 5 isolates in this study provide evidence that colony morphology variation represents heterogeneous phenotypes of *B. pseudomallei *with different fitness advantages to interact, survive and replicate in the presence of bactericidal substances within human macrophages.

A limitation of this study is that the experimental methods were laborious and time consuming, which restricted the number of strains we could examine. It is also unclear whether these *in vitro *assays using a human macrophage cell line are a good model for human infection. Further studies are required to determine the molecular mechanism of morphotype switching, and whether this is associated with persistence of *B. pseudomallei *in the human host.

## Conclusions

*B. pseudomallei *can produce different colony morphologies *in vivo *and *in vitro*. This study has described the intracellular survival and replication of two isogenic morphotypes II and III generated from 5 different parental type I *B. pseudomallei *in the U937 human macrophage cell line, and has examined the survival of these isogenic morphotypes compared to the parental types in the presence of a variety of substances and under conditions which are potentially encountered within the macrophage milieu. Data for 5 isolates demonstrated that there was variability in bacterial survival and replication following uptake by human macrophages between parental type I and types II or III, as well as variability between strains. Uptake of type III alone was associated with colony morphology switching. Type I was associated with survival in the presence of H_2_O_2_. In contrast, isogenic morphotype III demonstrated higher resistance to antimicrobial peptide LL-37. Specific morphotypes were not associated with survival with susceptibility to acid, acidified sodium nitrite, or resistance to lysozyme, lactoferrin, HNP-1 or HBD-2. Incubation under anaerobic conditions was a strong driver for switching of type III to an alternative morphotype in all isolates.

## Methods

### Bacterial isolates and isolation of isogenic morphotypes

Five *B. pseudomallei *isolates were examined in this study. Isolates 153, 164 and the reference isolate K96243 were cultured from cases of human melioidosis in Thailand, and isolates B3 and B4 were cultured from uncultivated land in northeast Thailand [19]. The colony morphology of all five parental isolates was type I, and isogenic types II and III were generated from type I of each strain using nutritional limitation [11]. Briefly, a single colony of type I on Ashdown agar was inoculated into 3 ml of TSB and incubated at 37°C in air in static conditions for 21 days. Bacterial culture was diluted and spread plated onto Ashdown agar. Morphotypes were identified using a morphotyping algorithm [11]. Isogenic types II and III generated from each parental type I were isolated from the plates of each strain.

### Growth curve analysis

Growth curves were performed for the 3 isogenic morphotypes of each of the 5 *B. pseudomallei *isolates. A colony of *B. pseudomallei *was suspended in sterile phosphate buffered saline (PBS). The bacterial suspension was adjusted to an optical density (OD) at 600 nm of 0.15 and diluted 100 times. One hundred microlitres of bacterial suspension was added to 10 ml of TSB and incubated at 37°C in air with shaking at 200 rpm for 28 h. At 2 h intervals, 100 μl of bacterial culture was removed, serially diluted 10-fold in PBS, and the bacterial count determined by plating on Ashdown agar in duplicate and performing a colony count following incubation at 37°C in air for 4 days. Doubling time was calculated.

### Cell line and culture conditions

Human monocyte-like cell line U937 (ATCC CRL-1593.2) originating from a histiocytic lymphoma was maintained in RPMI 1640 (Invitrogen) supplemented with 10% heat-inactivated fetal bovine serum (PAA Laboratories), 100 units/ml of penicillin and 100 μg/ml of streptomycin (Invitrogen) and cultured at 37°C in a 5% CO_2 _humidified incubator [20]. Before exposure to *B. pseudomallei*, 1 × 10^5 ^U937 cells per well were transferred to a 24 well-tissue culture plate (BD Falcon) and activated by the addition of 50 ng/ml of phorbol 12-myristate 13-acetate (PMA) (Sigma) over 2 days [20]. The medium was then replaced with 1 ml of fresh medium without PMA and incubated for 1 day. The differentiated macrophage was assessed by macrophage-like morphology [21]. Following washing 3 times with 1 ml of Hank's balance salt solution (HBSS) (Sigma), 1 ml of fresh medium was gently added to the macrophages.

### Interaction of *B. pseudomallei *isogenic morphotypes with human macrophages

The interaction assay was performed as previously described [11]. *B. pseudomallei *from an overnight culture on Ashdown agar was suspended in PBS, the bacterial concentration adjusted using OD at 600 nm and then diluted in PBS and inoculated into wells containing differentiated U937 cells to obtain an MOI of approximately 25 bacteria per cell. The MOI was verified by colony counting on Ashdown agar. Infected U937 cells were incubated at 37°C in 5% CO_2 _for 2 h. Non-adherent bacteria were removed by washing gently 3 times with 1 ml of PBS. The U937 cells were lysed with 1 ml of 0.1% Triton X-100 (Sigma), and the cell lysates serially diluted in PBS and spread plated on Ashdown agar to obtain the bacterial count. Colony morphology was observed [11]. The percentage of bacteria that were cell-associated was calculated by (number of associated bacteria × 100)/number of bacteria in the inoculum. The experiment was performed in duplicate for 2 independent experiments.

Intracellular survival and multiplication of *B. pseudomallei *in human macrophages were determined at a series of time points following the initial co-culture described above of differentiated U937 with *B. pseudomallei *for 2 h. Following removal of extracellular bacteria and washing 3 times with PBS, medium containing 250 μg/ml kanamycin (Invitrogen) was added and incubated for a further 2 h (4 h time point). New medium containing 20 μg/ml kanamycin was then added to inhibit overgrowth by any remaining extracellular bacteria at further time points. Intracellular bacteria were determined at 4, 6 and 8 h after initial inoculation. Infected cells were washed, lysed and plated as above. Intracellular survival and multiplication of *B. pseudomallei *based on counts from cell lysates were presented. Percent intracellular bacteria was calculated by (number of intracellular bacteria at 4 h) × 100/number of bacteria in the inoculum. Percent intracellular replication was calculated by (number of intracellular bacteria at 6 or 8 h × 100)/number of intracellular bacteria at 4 h. The experiment was performed in duplicate for 2 independent experiments.

### Growth in acid conditions

*B. pseudomallei *from an overnight culture on Ashdown agar was suspended in PBS and adjusted using OD at 600 nm to a concentration of 1 × 10^6 ^CFU/ml in PBS. Thirty microlitres of bacterial suspension was inoculated into 3 ml of Luria-Bertani (LB) broth at a pH 4.0, 4.5 or 5.0. The broth was adjusted to acid pH with HCl. Growth in LB broth at pH 7.0 was used as a control. The culture was incubated at 37°C in air with shaking at 200 rpm. At 1, 3, 6, 12 and 24 h time intervals, the culture was aliquoted and viability and growth determined by serial dilution and plating on Ashdown agar.

### Susceptibility of *B. pseudomallei *to reactive oxygen intermediates (ROI)

The sensitivity of *B. pseudomallei *to reactive oxygen intermediates was determined by growth on oxidant agar plates and in broth containing H_2_O_2_. Assays on agar plates were performed as described previously [22], with some modifications. Briefly, an overnight culture of *B. pseudomallei *harvested from Ashdown agar was suspended in PBS and the bacterial concentration adjusted using OD at 600 nm. A serial dilution of the inoculum was spread plated onto Ashdown agar to confirm the bacterial count and colony morphology. Ten microlitres of serial dilutions of bacteria in PBS were spotted onto LB agar containing 0, 170, 310, 625, 1,250 and 2,500 μM H_2_O_2_. Colony counts were performed after incubation at 37°C in air for 24 h. The number of colonies on plates containing H_2_O_2 _was compared with that on control plates and presented as bacterial survival (%). The assay was performed for 4 independent experiments.

Sensitivity to killing by hydrogen peroxide was further examined in LB broth. An overnight culture of *B. pseudomallei *on Ashdown agar was suspended in PBS and adjusted to approximately 1 × 10^8 ^CFU/ml. Ten microlitres of bacterial suspension was added into 1 ml of LB broth containing two-fold decreasing concentrations of H_2_O_2 _ranging from 500 to 31.25 μM. The mixtures were statically incubated at 37°C in air for 24 h and then the viable count and colony morphotype were determined by serial dilution and plating on Ashdown agar. The experiment was performed for 2 independent experiments.

### Susceptibility of *B. pseudomallei *to reactive nitrogen intermediates (RNI)

*B. pseudomallei *from an overnight culture on Ashdown agar was suspended in PBS and the bacterial concentration adjusted using OD at 600 nm. Thirty microlitres of bacterial suspension was added into 3 ml of two-fold decreasing concentrations of sodium nitrite (ranging from 10 to 0.1 mM) in LB broth at pH 5.0. The mixture was incubated at 37°C in air with shaking at 200 rpm and viable bacteria were determined at 6 h by serial dilution and plating on Ashdown agar. The number of viable bacteria in the presence of NaNO_2 _was compared with the number of bacteria in the inoculum and presented as bacterial survival (%). The experiment was performed in duplicate for 2 independent experiments.

### Susceptibility of *B. pseudomallei *to lysozyme and lactoferrin

*B. pseudomallei *cultured overnight on Ashdown agar was harvested and suspended in 10 mM Tris-HCl buffer pH 5.0 [23]. The bacterial suspension was adjusted to a concentration of 1 × 10^7 ^CFU/ml. Fifty microlitres of bacterial suspension was added to an equal volume of 400 μg/ml chicken egg white lysozyme (48,000 U/mg protein) (Sigma) to obtain a final concentration of 200 μg/ml. The mixture was incubated at 37°C in air for 24 h, after which 10 μl of 10-fold serial dilutions were dropped on Ashdown agar. Sensitivity to lysozyme was also tested in the presence of 3 mg/ml lactoferrin (Sigma) in a separate experiment [23]. *E. coli *strain HB101 was tested in parallel as a control.

### Susceptibility to human α-defensin and β-defensin

*B. pseudomallei *was tested for resistance to HNP-1 and HBD-2 (Peptide international) as described previously [24], with the exception that HNP-1 was used at twice the dose. *E. coli *strain HB101 was tested in parallel as a control. Briefly, *B. pseudomallei *or *E. coli *strain HB101 colonies were washed and suspended in 1 mM sodium phosphate buffer pH 7.4 containing 1% TSB [24]. The bacterial suspension was adjusted to a concentration of 1 × 10^7 ^CFU/ml. Twenty microlitres of bacterial suspension was mixed with an equal volume of 200 μg/ml HNP-1 or HBD-2 to obtain a final concentration of 100 μg/ml antimicrobial peptide and incubated at 37°C in air for 3 h. The viable bacterial count was determined by dropping a 10-fold serial dilution on Ashdown agar.

### Susceptibility to antimicrobial activity of human cathelicidin

*B. pseudomallei *susceptibility to cathelicidin LL-37 was tested using a microdilution method [25]. LL-37 was kindly provided by Dr. Suwimol Taweechaisupapong, Department of Oral Diagnosis, Faculty of Dentistry, Khon Kaen University and Dr. Jan G.M. Bolscher, Department of Oral Biochemistry, Van der Boechorststraat, Amsterdam, The Netherlands. A loop of bacteria was washed 3 times in 1 mM potassium phosphate buffer (PPB) pH 7.4 and suspended in the same buffer. The bacterial suspension was adjusted to a concentration of 1 × 10^7 ^CFU/ml. Fifty microlitres of suspension was added into wells containing 50 μl of a 2-fold serial dilution of human cathelicidin in PPB (to obtain a final concentration of 3.125-100 μM), The mixture was incubated at 37°C in air for 6 h and viability of bacteria was determined by plating a 10-fold serial dilution on Ashdown agar. The assay was performed in duplicate.

### Growth in low oxygen and anaerobic conditions

An overnight culture of *B. pseudomallei *on Ashdown agar was suspended in PBS and adjusted to a concentration of 1 × 10^8 ^CFU/ml. The bacterial suspension was 10-fold serially diluted and 100 μl spread plated on Ashdown agar to obtain approximately 100 colonies per plate. Three sets of plates were prepared per isolate and incubated separately at 37°C in 3 conditions: (i) in air for 4 days (control); (ii) in an GasPak EZ Campy Pouch System to produce an atmosphere containing approximately 5-15% oxygen (BD) for 2 weeks; or (iii) in an anaerobic jar (Oxoid) with an O_2 _absorber (AnaeroPack; MGC) for 2 weeks and then re-exposed to air at 37°C for 4 days. The mean colony count was determined for each morphotype from 5 *B. pseudomallei *isolates after incubating bacteria in air for 4 days (control). % colony count for each isolate incubated in 5-15% oxygen or in an anaerobic jar for 14 days was calculated in relation to the colony count of the control incubating bacteria in air for 4 days.

### Colony morphology switching

Seven conditions were observed for an effect on morphotype switching, as follows: (i) culture in TSB in air with shaking for 28 h, (ii) intracellular growth in macrophage cell line for 8 h, (iii) exposure to 62.5 μM H_2_O_2 _in LB broth for 24 h, (iv) growth in LB broth at pH 4.5 for 24 h, (v) exposure to 2 mM NaNO_2 _for 6 h, (vi) 6.25 μM LL-37 for 6 h, and (vii) incubation in anaerobic condition for 2 weeks and then re-exposure to air for 4 days. All experiments were performed using the experimental details described above. *B. pseudomallei *morphotype on Ashdown agar following incubation in air at 37°C for 4 days was defined and compared with the starting morphotype. Morphotype switching was presented as the proportion (%) of alternative types in relation to the total colonies present. Assays of resistance to HNP-1, HBD-2, lysozyme and lactoferrin employed a drop method to assess bacterial survival and colony morphology could not be accurately determined.

### Statistical analysis

Statistical analysis was performed using the statistical program STATA version 10.1. Log transformation of continuous dependent variables was performed as appropriate. Nested repeated measures ANOVA was used to test continuous dependent variables between 3 isogenic morphotypes. A difference between 3 morphotypes was considered to be statistically significant when the *P *value was less than or equal to 0.05, after which pairwise comparisons were performed between each morphotype. All *P *values for pairwise analyses were corrected using the Benjamini-Hochberg method for multiple comparisons [26].

## Authors' contributions

ST carried out the experiments and data analysis. AT isolated and maintained isogenic morphotypes. DL participated in statistical analysis. SK and ND provided materials and intellectual comments. SJP participated in the design of the study, and assisted in the writing of the manuscript. NC participated in the design of the study, data analysis and coordination and writing of the manuscript. All authors read and approved the final manuscript.
